# Estimating Simultaneous Equation Models through an Entropy-Based Incremental Variational Bayes Learning Algorithm

**DOI:** 10.3390/e23040384

**Published:** 2021-03-24

**Authors:** Rocío Hernández-Sanjaime, Martín González, Antonio Peñalver, Jose J. López-Espín

**Affiliations:** Center of Operations Research, Miguel Hernández University, 03202 Elche, Spain; martin.gonzaleze@umh.es (M.G.); a.penalver@umh.es (A.P.); jlopez@umh.es (J.J.L.-E.)

**Keywords:** computational econometrics, simultaneous equation model, clustering, variational algorithms, Shannon entropy, Leonenko estimator

## Abstract

The presence of unaccounted heterogeneity in simultaneous equation models (SEMs) is frequently problematic in many real-life applications. Under the usual assumption of homogeneity, the model can be seriously misspecified, and it can potentially induce an important bias in the parameter estimates. This paper focuses on SEMs in which data are heterogeneous and tend to form clustering structures in the endogenous-variable dataset. Because the identification of different clusters is not straightforward, a two-step strategy that first forms groups among the endogenous observations and then uses the standard simultaneous equation scheme is provided. Methodologically, the proposed approach is based on a variational Bayes learning algorithm and does not need to be executed for varying numbers of groups in order to identify the one that adequately fits the data. We describe the statistical theory, evaluate the performance of the suggested algorithm by using simulated data, and apply the two-step method to a macroeconomic problem.

## 1. Introduction

Simultaneous equation models (SEMs) constitute the reference statistical methodology in the analysis of jointly dependent variables [1]. Most applications are primarily found in econometrics [2,3], but also in medicine [4] and even in the study of divorce rates [5]. These models can be seen as multivariate regression models that reflect the simultaneity in structural relations in a system of multiple endogenous variables. Traditionally, it is common to assume homogeneity in the structural relations across observations and to estimate a single set of structural parameters. However, many practical situations in a wide range of disciplines (e.g., economics, finance, marketing, or sociology) involve structural changes in the studied variables or unobserved heterogeneity in data. Consequently, this simplification is often unrealistic and likely to produce misleading results.

Over the past few years, the problem of heterogeneity in data was studied for regression models. Many papers in the social sciences modelled anomalies by segmented regression. In this context, some aspects of change were investigated through formal statistical testing procedures [6,7]. Alternatively, structural-break inference was discussed using information criterion methods based on modifications of the Schwarz criterion (1978; BIC) [8,9], the Akaike criterion (1974; AIC) [10], and, more recently, the penalty term of several information criteria [11]. In particular, these models were widely applied in macroeconomics, where government interventions and policy changes at specific time points can affect both economic and market structures; examples include the analysis of regional stock exchanges in the United States [12] or the Euro area monetary policy [11]. Nevertheless, segmented regression also arises in a natural way in the context of industrial chemistry [13], agricultural and biological sciences [14], or climatology [15].

In the structural modelling framework, which embeds simultaneous equation models, researchers are also prone to estimating the model as if data belong to a single population [16]. However, this assumption does not always hold, and several authors cautioned against pooling data that may come from different segments [17,18]. Furthermore, traditional fit statistics do not alert about the presence of unaccounted heterogeneity in the model. Problems stemmed from failure to handle heterogeneity in structural equation models are illustrated in Jedidi et al. [19].

Typically, the procedure used to overcome these statistical difficulties is denominated multigroup structural equation modelling [20,21]. This supposes that the sample can be partitioned into *G* groups (also referred to as kernels from now on), assumed to be known a priori, and it estimates separate structural equation models for each of the *G* groups. In principle, models of different forms can be specified for each of the *G* groups. Nevertheless, the most relevant drawback of this a priori segmentation approach is that, in many situations, researchers do not know the number of groups that account for heterogeneity and do not have enough information to form segments a priori.

Thus, groups have to be determined from the data post hoc, and different strategies are possible. One option is to implement the finite-mixture structural equation model (STEMM) developed in Jedidi et al. [22] for simultaneously detecting and treating unobserved heterogeneity via the expectation-maximisation (EM) algorithm. This model generalises the multigroup structural equation model to the case in which group membership cannot be established a priori. In particular, it encompasses several specialised models, including finite mixture simultaneous equation models [19,23]. Alternatively, a sequential two-step process that first forms groups using some clustering algorithm applied to all variables, and then implements the multigroup structural equation modelling methodology to estimate each of the resulting groups can be considered. Note that for either approach, the numbers of groups must be prespecified when running the procedure, and this information is usually unknown. Methodologically, the finite-mixture model is robust and superior in goodness of fit to sequential data analysis strategies. However, one must consider that the clustering algorithm commonly used in the two-step scheme is K-means, which is not satisfactory, especially when the groups significantly overlap. Therefore, a two-step approach may be problematic [24,25], but its performance heavily depends on the type of clustering algorithm used in the first step.

Clustering procedures have two significant downsides. First, most clustering algorithms struggle with high-dimensional data. This weakness is recognised in the literature as the curse of dimensionality [26]. Data-reduction methods (e.g., principal component analysis) were discussed in the context of clustering and structural equation modelling [27,28]. Nevertheless, in this work, we restricted dimensions to a manageable number. Second, a caveat is that very large samples are required to perform cluster analysis. In the structural equation model framework, if the sample size is modest, the researcher may have no choice but to use other approaches such as the MIMIC method [16] or the STEMM approach. However, in many studies, we expect the sample size to be reasonably large.

This paper proposes a two-step procedure that in the first step picks the appropriate number of groups and classifies observations using an entropy-based incremental variational Bayes (EBIVB) algorithm. Traditionally, the two-step strategy used in the literature when struggling with heterogenous observations includes the standard K-means algorithm in the first step, which is a nonhierarchical distance-based algorithm. Unlike this approach, we used an entropy-based hierarchical clustering. There are two main advantages in our proposal. First, the number of clusters is not fixed and does not need to be specified by the researcher. Second, the use of entropy as the similarity measurement in the clustering step avoids distance calculation, reducing the outlier effect on cluster quality. Moreover, previous studies in the structural equation modelling context analysed the robustness of different model selection criteria (e.g., CAIC, BIC) in choosing the correct number of groups. To our knowledge, this study is the first using a clustering algorithm that identifies the unknown groups based on the Gaussian deficiency (GD). On the whole, these factors are expected to improve model estimation. The rest of the paper is divided as follows: Section 2 provides a brief overview of the used statistical model and clustering algorithm. In Section 3, the two-step method is tested in a simulation study, and the obtained results are compared with other sequential approaches. Additionally, performance comparisons on real data are illustrated in Section 4. Lastly, main conclusions and future work are listed in Section 5.

## 2. Methodology

### 2.1. Simultaneous Equation Models (SEMs)

Simultaneous equation models consist of a system of linear regression equations with jointly dependent variables. In SEMs, variables can be classified as (i) endogenous if they are explained through a set of variables, i.e., dependent variables; or (ii) predetermined if they are independent nonrandom variables, i.e., exogenous and lagged endogenous variables. The main property of a simultaneous equation model is that the model allows for endogenous variables to be incorporated as explanatory variables in other equations. This way, SEMs contemplate the interdependent relations across variables. Formally, the structural form of the model for a system with *m* equations, *m* endogenous variables, and *k* predetermined variables is

(1) Y=YA+XB+U,

where $Y=[y^{1},\cdots ,y^{m}]$ is a N×m matrix of *N* observations of *m* endogenous variables, $X=[x^{1},\cdots ,x^{k}]$ is a N×k matrix of *N* observations of *k* predetermined variables and $U=[u^{1},\cdots ,u^{m}]$ is a N×m matrix of the structural disturbances of the system. Matrices *A* (m×m) and *B* (k×m) are the endogenous and exogenous unknown coefficient matrices, respectively (by convention, $a_{ii}=0$, i=1,2,⋯,m).

The error terms $u_{t\cdot }\;(t=1,\cdots ,N$) are assumed to be normally distributed:

(2) $$\begin{array}{ccc} & u_{t\cdot }^{\prime }∼N\left(0,\Sigma \right),\;E\left(u_{t\cdot }^{\prime },u_{t^{\prime }\cdot }\right)=\delta_{tt^{\prime }}\Sigma \; & t,t^{\prime }=1,2,\cdots ,N \end{array}$$

where $\delta_{tt^{\prime }}$ is the Kronecker delta and Σ a positive definite matrix.

Moreover, we assume that error terms are uncorrelated with the predetermined variables of the system, and that there is no linear dependence among the predetermined variables. Lastly, assuming that (I−A) is nonsingular, the reduced form of the system is given by

(3) $$\begin{array}{c} Y=X\Pi +V, \end{array}$$

where $\Pi =B{\left(I-A\right)}^{-1}$, $V=U{\left(I-A\right)}^{-1}$.

The random vectors $v_{t\cdot }$ have the following properties:

(4) $$\begin{array}{cc} & v_{t\cdot }^{\prime }∼N\left(0,\Omega \right),\;v_{t\cdot }=u_{t\cdot }{\left(I-A\right)}^{-1},\;\Omega ={\left({\left(I-A\right)}^{\prime }\right)}^{-1}\Sigma {\left(I-A\right)}^{-1},\\ & Cov\left(v_{t\cdot }^{\prime },v_{t^{\prime }\cdot }\right)=\delta_{tt^{\prime }}\Omega \;t,t^{\prime }=1,2,\cdots ,N \end{array}$$

In SEMs, parameter estimation can only be accomplished when the system is identified. An equation *i* (i=1,⋯,m) is identified if the order condition is fulfilled, i.e., $m_{i}-1\leq k-k_{i}$ where $m_{i}$ and $k_{i}$ are the number of endogenous and exogenous variables in equation *i*, respectively [29]. Then, if the model can be estimated, indirect least-squares (ILS), two-stage least-squares (2SLS), three-stage least-squares (3SLS), or maximum-likelihood (ML) methods are the customary calculation approaches [30].

The goodness of the model can be assessed by evaluating diverse global measures of fit, such as information criteria. The Akaike information criterion (AIC); its corrected version AICc [31]; or the Bayesian information criterion (BIC) were adapted to SEMs [32]. Here, AIC was chosen for a SEM, expressed as follows.

(5) $$AIC=N\ln|\hat{\Sigma_{e}}|+2\sum_{i=1}^{m}\left(m_{i}+k_{i}-1\right)+m\left(m+1\right),$$

where $|\hat{\Sigma_{e}}|$ is the determinant of error covariance matrix $e_{i}$ (i=1,⋯,m) and $e_{i}$ the difference between $y_{i}$ and its estimation given equation *i*.

If the information criterion of one model is lower than the information criterion of another, the former model is considered better than the second. Because AIC is based on the maximum-likelihood method, the covariance matrix of the prediction errors has to be minimised.

### 2.2. Entropy-Based Incremental Variational Bayes Learning Algorithm (EBIVB)

Gaussian mixture models are frequently used as a formal approach to clustering [33]. These statistical pattern-recognition techniques were designed to deal with complex probability density functions, and those based on Gaussian kernels are especially useful for modelling data when forming clustering structures as being generated by a set of different kernels. In this latter approach, the estimation of the parameters of each kernel can be carried out by using different methods: maximum likelihood (ML), maximum a posteriori (MAP), or Bayesian inference.

The third estimation option is based on a fully Bayesian inference model [34]. This method can be computationally demanding and may involve intractable integrals as the complexity of the model increases. Thus, some alternatives emerged to overcome these downsides: the Laplacian method [35], Markov chain Monte Carlo (MCMC) [36], and variational methods [37].

In this paper, we use an incremental extension of the variational Bayes (VB) method first introduced in Peñalver and Escolano [38]. This is an iterative procedure that starts with only one kernel (K=1), which is initially provided by the sample, and at each iteration incorporates a new component into the mixture by splitting one of the current kernels.

Formally, given a dataset $X=\{x_{1},\ldots ,x_{N}\}$ of *N* observations, mixture distribution can be interpreted as a latent variable model that for each observation $x_{n}$ introduces a set of binary latent variables describing which kernel from the mixture generated the observation, $z_{in}\in \left\{0,1\right\}$ where i=1,…,K, and *K* is the number of kernels. In this way, $z_{in}=1\Leftrightarrow$ component *i* gave rise to observation $x_{n}$ and $\sum_{i=1}^{K}z_{in}=1$.

The conditional probability density function of observations *X* given $z=\{z_{in}\}$ is normal with mean $\mu_{i}$ and inverse covariance matrix $T_{i}$, stated as $P\left(X|\mu ,T,z\right)=\prod_{n=1}^{N}\prod_{i=1}^{K}N{\left(x_{n}|\mu_{i},T_{i}\right)}^{z_{in}}$, where the prior distribution of *z* is the product of multinomials $P\left(z|\pi \right)=\prod_{i=1}^{K}\prod_{n=1}^{N}\pi_{i}^{z_{in}}$. In addition, the observations are assumed to be independently generated, and the conjugate priors over the means and inverse covariances are, respectively, $P\left(\mu \right)=\prod_{i=1}^{K}N\left(\mu_{i}|0,\beta I\right)$ and $\;P\left(T\right)=\prod_{i=1}^{K}W\left(\nu ,V\right)$, where β is a small fixed parameter corresponding to a wide prior over μ, *I* is the identity matrix, W represents Wishart distribution, and *V* and ν stand for the scale matrix and the degrees of freedom for a wide prior over *T*. Lastly, the joint distribution is specified as

(6) P(X,μ,T,z|π)=P(X|μ,T,z)P(z|π)P(μ)P(T)

The objective is to optimise mixing coefficients π by maximising data marginal likelihood. This is analytically unfeasible, but variational methods [39] can provide a lower bound of P(X,μ,T,z|π) by introducing a distribution Q(Θ) in the log marginal likelihood expression:

(7) $$\begin{array}{cc} \log P(X|\pi ) & =\log\sum_{z}\int P\left(X,\Theta |\pi \right)d\Theta =\log\sum_{z}\int Q\left(\Theta \right)\frac{P(X,\Theta |\pi )}{Q(\Theta )}d\Theta \\ \; & \geq \sum_{z}\int Q\left(\Theta \right)\log\frac{P(X,\Theta |\pi )}{Q(\Theta )}d\Theta =L\left(Q\right) \end{array}$$

where Θ={μ,T,z} to simplify notation.

Because the true log likelihood is independent of Q, we simply need to maximise the lower bound of the true marginal likelihood. If a mean-field approximation [39,40] is adopted, then Q can be factorised over the variables in Θ; so,

(8) $$Q\left(\Theta \right)=Q_{z}\left(z\right)Q_{\mu }\left(\mu \right)Q_{T}\left(T\right)$$

Starting from the expressions for the joint distribution in (6), the lower bound in (7), the factorisation in (8) and the equations for the variational factors as described in Peñalver and Escolano [38], the lower bound L(Q) can be evaluated as

(9) $$\begin{array}{ccc} L(Q) & = & \langle \log P(X|\mu ,T,z)\rangle +\langle \log P(z)\rangle +\langle \log P(\mu )\rangle +\langle \log P(T)\rangle \\ & & -\langle \log Q_{z}\left(z\right)\rangle -\langle \log Q_{\mu }\left(\mu \right)\rangle -\langle \log Q_{T}\left(T\right)\rangle \end{array}$$

The estimation of the mixing coefficients in the mixture is obtained by the maximisation of the bound in (9) with respect to π and an expectation-maximisation procedure is needed. After each EM iteration, bound L(Q) should not decrease. This fact and the possible convergence of some coefficients to zero are used both as model order selection and stopping criteria.

At each iteration, this EBIVB approach compares the entropy of the underlying probability density function of each kernel in the mixture regarding the theoretical entropy of a Gaussian [41], and the worst component in terms of its Gaussian deficiency (GD) is selected. Therefore, this component is supposed to be the one with greater entropy difference with a true Gaussian. Then, the worst-adjusted kernel is removed and replaced by two new kernels adequately separated from each other.

For the calculation of the GD of a component, it is necessary to estimate the entropy of that component. The Leonenko estimator [42] is a *k* neural-network (NN) entropy estimator based on Shannon entropy formula H(X)=−∫f(x)logf(x)dx, which may be interpreted as an average of the logf(x), being f(x) an existing probability density function.

In order to estimate H(X), probability distribution $P_{k}\left(\epsilon \right)$ of the distance between a sample $x_{i}$ and its *k*-NN is examined. This way, if we consider a ball of diameter ϵ centred at $x_{i}$, and there is a point within distance ϵ/2, then there are k−1 other points closer to $x_{i}$, and N−k−1 points farther from it.

(10) Pk(ϵ)dϵ=kN−1kdpi(ϵ)dϵpik−1(1−pi)N−k−1,

where $p_{i}$ is the mass of the ϵ-ball and $p_{i}\left(\epsilon \right)=\int_{||\xi -x_{i}\left|\right|<\epsilon /2}f\left(\xi \right)d\xi$.

The expectation of $\log p_{i}\left(\epsilon \right)$ is expressed as

(11) $$\begin{array}{ccc} E(\log p_{i}) & = & \int_{0}^{\infty }P_{k}\left(\epsilon \right)\log p_{i}\left(\epsilon \right)d\epsilon =\psi \left(k\right)-\psi \left(N\right), \end{array}$$

where ψ(·) is the digamma function.

If f(x) is assumed to be constant all over the ϵ-ball, we can consider approximation $p_{i}\left(\epsilon \right)\approx \frac{V_{d}}{2^{d}}\epsilon^{d}\mu \left(x_{i}\right)$, where *d* is the dimension, and $V_{d}$ is the volume of the unit ball B(0,1), defined as $V_{d}=\frac{\pi^{\frac{d}{2}}}{\Gamma (\frac{d}{2}+1)}$. Then, approximation $\log f\left(\epsilon \right)\approx \psi \left(k\right)-\psi \left(N\right)-dE\left(\log\epsilon \right)-\log\frac{V_{d}}{2^{d}}$ can be formulated, and lastly, the estimation of H(X) is given by

(12) $$\hat{H}\left(X\right)=\psi \left(N\right)-\psi \left(k\right)+\log\frac{V_{d}}{2^{d}}+\frac{d}{N}\sum_{i=1}^{N}\log\epsilon_{i}$$

where $\epsilon_{i}=2||x_{i}-x_{j}\left|\right|$ is twice the distance between sample $x_{i}$ and its *k*-NN $x_{j}$.

Once the worst component is selected and superseded by two new ones, a new standard step of the VBgmm method [43] with K+1 components is conducted to maximise the marginal likelihood for the updated number of components. This process is repeated until convergence is reached. The split fails when the new component does not provide a better fit to the data, and some of the mixing coefficients tend to zero. In this case, it is eliminated, and the algorithm ends with a mixture of *K* kernels instead of K+1.

The split proceeding is an ill-posed problem since it may have more than one solution that discontinuously depends upon the initial data. Furthermore, a new EM-like method is needed every time a split test is completed. Therefore, we ensure that the number of splits are controlled, and a particular case of the procedure described in Dellaportas and Papageorgiou [44] is used with that purpose. The overall process is linear with the number of kernels (one split per iteration) and not prone to initialisation. This fact accelerates convergence and prevents the algorithm from falling into a local maximum of the marginal likelihood function, improving the performance of other current variational methods.

## 3. Experiment Results

We conducted two simulation experiments to test the performance of the proposed two-step estimation strategy in handling heterogeneity for correctly specified simultaneous equation models. The first experiment compares the Akaike information criterion of different estimated models: aggregate analysis (AGG), which ignores heterogeneity; known group membership (GM); and percentage model (PM), which classifies a proportion p% of the observations in an incorrect group deliberately. The purpose of the second experiment is to study the behaviour of the solution estimates of the suggested sequential method when varying the number of groups in exactly identified and overidentified SEM, assuming that the distributional form is properly specified.

Four different values for endogenous variables were examined, *m* = 2, 4, 6, and 8, and the number of exogenous variables was fixed to k=10. To approximate real-life applications, we used a sufficiently large random sample of 1000 observations per group in all simulations. This fixed sample size was generated assuming that data in each group have a multivariate normal distribution and the clusters were constructed to be either non overlapping or slightly overlapping. The next section describes in detail the data-generation process in the simulation studies. For each problem size, we performed 10 replications to reduce the chance of outliers.

Experiments were run in a parallel NUMA node with 4 Intel hexacore Nehalem-EX EC E7530 with 24 cores at 1.87 GHz and 32 GB of RAM. All tests were implemented in C code with the exception of the data-generation process and the clustering algorithm. R statistical package GNU R version 3.5.2. was used in the data simulation, and MATLAB library R2016b in the clustering procedure.

### 3.1. Data-Generation Process

Consider the simultaneous equation model (1) rewritten as follows:

(13) $$YA^{*}+XB+U=0,$$

where Y,X,B, and *U* correspond to the matrices described in (1), and $A^{*}=\left(A-I\right)$ has each main diagonal entry set to −1, so that each equation contains its main endogenous variable. Consider the observations of the endogenous (*Y*) and predetermined (*X*) variables clustered into *G* groups, that is,

$$Y=\left[\begin{array}{c} Y_{1}\\ Y_{2}\\ \vdots \\ Y_{G} \end{array}\right]\in R^{N\times m}\;X=\left[\begin{array}{c} X_{1}\\ X_{2}\\ \vdots \\ X_{G} \end{array}\right]\in R^{N\times k}$$

In all experiments, the simultaneous equation model was constructed to satisfy the order condition, rejecting any codification that led to an underidentified model. This procedure ensured the identification of the system. For the structural model, each element in matrices $A^{*}$ and *B* was randomly generated by following a uniform distribution over the interval from −10 to 10. The same coefficient matrices, $A^{*}$ and *B*, were considered for all groups in the data-generation process, thereby assuming the model was initially the same across groups. However, once established, the model may be disrupted by unpredictable external events such as government intervention in the economy or changes in variables resulting from accidents or disasters.

In order to reflect changes due to unexpected exogenous factors that affect the endogenous variables, we directly induced shocks to the endogenous variables in the generation process. To this end, the sample data of the endogenous variables were created for *G* groups by varying the mean and dispersion parameters for each group. Hence, we considered the data in each of the clusters as the response of the set of endogenous variables at the time of a specific shock and at subsequent times. To avoid undersized samples, we set a fixed sample size of 1000 observations per group in all simulations. Thus, we also provided a sufficiently large sample size for the clustering algorithm. The endogenous variables were assumed to have multivariate normal distribution. For each group, g ∈G, the mean of each endogenous variable was randomly chosen from a uniform distribution from −8 to 8 for m=2, and −10 to 10 in the rest of the cases. The elements of the variance–covariance matrix of the endogenous variables were also randomly selected from uniform distribution from −8 to 8, −10 to 10, −15 to 15, and −20 to 20 for m=2,4,6,8, respectively. These ranges were chosen to allow for some degree of separation among groups. The objective was to create nonoverlapping or slightly overlapping clusters. We computed rmvnorm in R to generate random values from multivariate normal distribution, which constituted the observations of each of the groups.

In all experiments, once the coefficient matrices and endogenous variables had been generated, the exogenous variables were calculated. To this effect, we considered the QR decomposition of exogenous coefficient matrix *B*$\in R^{k\times m}$, with k≥m:

(14) B=QR,

where $Q\in R^{k\times m}$ with $Q^{T}Q=I_{m}$ and $R\in R^{m\times m}$ is an upper triangular matrix.

Using the QR decomposition of *B*, and assuming rank(B)=n, so that *R* is nonsingular:

(15) $$\begin{array}{c} -YA^{*}R^{-1}=XQ=X\left[\begin{array}{c} Q_{1}\\ Q_{2} \end{array}\right], \end{array}$$

where $Q_{1}\in R^{m\times m}$ and $Q_{2}\in R^{(k-m)\times m}.$

Similarly, *X* could be partitioned into two submatrices, $X=\left[\begin{array}{c} X_{1}\;X_{2} \end{array}\right]$ where $X_{1}\in R^{N\times m}$ and $X_{2}\in R^{N\times (k-m)}$, and structural form (13) can be expressed as

$$-YA^{*}R^{-1}=\left[\begin{array}{c} X_{1}\;X_{2} \end{array}\right]\left[\begin{array}{c} Q_{1}\\ Q_{2} \end{array}\right]=X_{1}Q_{1}+X_{2}Q_{2}$$

(16) $$X_{1}=-\left[YA^{*}R^{-1}+X_{2}Q_{2}\right]\;Q_{1}^{-1}$$

To obtain the sample data of the set of exogenous variables, matrix $X_{2}$ was randomly generated following a uniform distribution over the interval from 0.1 to 1.1, and $X_{1}$ was calculated by substituting in (16), assuming $Q_{1}$ was nonsingular.

### 3.2. Simulation Experiments

#### 3.2.1. First Experiment—Model Selection

This experiment compares the goodness of fit of different estimated models. Previous studies addressed the problem of unobserved heterogeneity in simultaneous equation models from two different approaches. One option is to estimate a single-group simultaneous equation model by using aggregate data. However, by doing so, one implicitly assumes data homogeneity. The second option is to theorise that there are different groups that could follow different models allowing for parameter values to differ across groups.

Under this second strategy, if group membership is known a priori, the researcher can use standard multigroup methods. The main limitation of this approach is that, in most situations, groups are unknown and have to be determined. To illustrate the bias introduced by two-step procedures that apply a clustering algorithm followed by multigroup simultaneous equation analysis, a percentage model was included in the experiment. This model considers the known group membership model as starting point and selects a percentage of total observations p% to be assigned to an incorrect group. These observations are chosen from among those in each group falling out of the ellipsoid of equal concentration associated with a probability of 0.75. For each group, the ellipsoid is determined by the corresponding group multivariate normal distribution [45]. Lastly, these observations are reallocated into the group that minimises the distance from the observation coordinates to the cluster centroids, without considering their group of origin.

In order to test the robustness of the proposed clustering algorithm (CA), in this first experiment we examined the performance of the aggregate model (AGG), the known group membership model (GM), and the percentage model (PM) where p=5%,10%, and 15%. Note that the known group membership model corresponds to the percentage model with p=0%. For ease of comparison, the known group membership (GM) model was considered to be the benchmark. Table 1 shows the experiment outcomes for the true number of groups *G* = 4.

Expectedly, the known group membership model outperformed aggregate and percentage analysis. In all cases, the aggregate model recorded the worst result. The AIC value decreased from the the percentage model with p=15% to the known group membership model, i.e., p=0%. Interestingly, the goodness of fit provided by the sequential analysis was between the known group membership model (GM) and the percentage model (PM) with p=5% for all cases, except for m=2 when the clustering configuration found by the algorithm enhanced the known group membership results. Therefore, the clustering classification accuracy was equivalent to the performance of the percentage model with p∈ (0, 0.05). Furthermore, the percentage of error in clustering classification decreased when the dimension of the problem determined by the number of endogenous variables increased.

#### 3.2.2. Second Experiment—Solution-Estimate Analysis

This experiment examines the performance of the clustering technique to select the optimal number of groups and to classify observations into the most accurate group. The principal goal is to determine the ability of the clustering algorithm to recover group membership. The proposed sequential analysis first applies the clustering algorithm to all endogenous variables, and a simultaneous equation model is then estimated within each of the resulting groups. Alternatively, we could have estimated the simultaneous equation model for different numbers of clusters and selected the model returning the best fit with regard to a given information criteria.

To assess the strength of the clustering algorithm in detecting the correct number of groups, we calculated the AIC value of some randomly selected problems. Table 2 shows the evolution of the Akaike information criterion from the aggregate model (G=1) up to the model estimated when the clustering algorithm stopped; in other words, up to the model with the optimal number of groups detected by the algorithm.

According to Table 2, for the selected problems, the clustering algorithm always stopped at G=4. On the basis of the computational results, the goodness of fit value provided by the two-step strategy enhanced the score of the one-population model (G=1). The Akaike information criteria improved from the aggregate model to the model estimated with the optimal number of clusters. Thus, the algorithm results were consistent with the evolution of the AIC. Overall, for G=4 being the true number of groups, the algorithm picked the same number of groups as the known group membership model (GM) in 100% of the runs. The success rate of the algorithm was stable when the number of endogenous variables increased. As a final remark, note that the algorithm could pick an optimal number of groups different from the number of groups corresponding to the true model if the resulting clustering configuration was more accurate or plausible than the one in the original model.

## 4. Empirical Application

This section presents an illustrative application of our method to Klein’s Model I. Klein [2] developed three Keynesian macroeconomic models to study the United States economy for the 1921–1941 period, and examine the consequences of different political measures. The smallest of these three models, known as Model I, describes the workings of the U.S. economy in terms of a simultaneous six-equation system: three behavioural equations, an equilibrium condition, and two accounting identities. The model may be written following the notation set out in Greene [46]:

$$\begin{array}{cc} C_{t} & =\alpha_{0}+\alpha_{1}P_{t}+\alpha_{2}P_{t-1}+\alpha_{3}\left(W_{t}^{p}+W_{t}^{g}\right)+\varepsilon_{1t}\\ I_{t} & =\beta_{0}+\beta_{1}P_{t}+\beta_{2}P_{t-1}+\beta_{3}K_{t-1}+\varepsilon_{2t}\\ W_{t}^{p} & =\gamma_{0}+\gamma_{1}X_{t}+\gamma_{2}X_{t-1}+\gamma_{3}A_{t}+\varepsilon_{3t}\\ X_{t} & =C_{t}+I_{t}+G_{t}\;\left(\mathrm{equilibrium}\mathrm{demand}\right)\\ P_{t} & =X_{t}-T_{t}-W_{t}^{p}\;\left(\mathrm{private}\mathrm{profits}\right)\\ K_{t} & =K_{t-1}+I_{t}\;\left(\mathrm{capital}\mathrm{stock}\right) \end{array}$$

where

$$\begin{array}{cc} C_{t} & =\mathrm{Consumption}\mathrm{expenditure}\\ I_{t} & =\mathrm{Investment}\\ P_{t} & =\mathrm{Private}\mathrm{profits}\\ K_{t} & =\mathrm{Capital}\mathrm{stock}\\ G_{t} & =\mathrm{Government}\mathrm{nonwage}\mathrm{spending}\\ T_{t} & =\mathrm{Indirect}\mathrm{business}\mathrm{taxes}\mathrm{plus}\mathrm{net}\mathrm{exports}\\ W_{t}^{g} & =\mathrm{Government}\mathrm{wages}\\ W_{t}^{p} & =\mathrm{Private}-\mathrm{sector}\mathrm{wages}\\ X_{t} & =\mathrm{Total}\mathrm{demand}\\ A_{t} & =\mathrm{Time}\mathrm{trend}\mathrm{measured}\mathrm{as}\mathrm{years}\mathrm{from}1931 \end{array}$$

In the above model, we have 6 endogenous variables ($C_{t}$, $I_{t}$, $P_{t}$, $W_{t}^{p}$, $X_{t}$, and $K_{t}$), 3 lagged endogenous variables ($P_{t-1}$, $X_{t-1}$ and $K_{t-1}$) and 4 exogenous variables, including the time trend ($G_{t}$, $T_{t}$, $W_{t}^{g}$ and $A_{t}$). The three former equations linearly describe the consumption, investment, and private-sector wage bill, respectively. Additionally, we have three identities that express the total demand according to all production undertaken in the economy, the profits net of taxes, and the capital stock in any period, respectively.

Whether the data are homogeneous or not, a widespread practice is to estimate a single set of aggregate-level structural parameters. However, such aggregate estimates are not fully acceptable if there is significant heterogeneity in the data because no variation in the structural relations is permitted.

To test for heterogeneity, the proposed two-step procedure was used to obtain estimates of the structural parameters for different numbers of groups beyond the aggregate case of G=1. For purposes of illustrating our algorithm, the application was conducted using time-series data from 1921 to 2000 in order to provide a reasonably sample size. All variables were measured in USD billions of 1996 [47] except for time $A_{t}$, measured in calendar years from 1961.

Moreover, to demonstrate the benefits of the proposed methodology, it was compared with other state-of-the-art algorithms, namely, it was contrasted with the two-step scheme that uses K-means as clustering algorithm. The resulting AIC values of the different approaches are shown in Table 3, departing from G=1 until the stop criterion explained in the preceding sections was reached.

Table 3 offers two main conclusions. First, the proposed algorithm stopped when the sample is divided into three groups, suggesting that G=3 is the optimal solution. Furthermore, the AIC criterion for model selection ($AIC_{CA}$) reinforced the choice of the three-group model provided by the stop criterion implemented in our methodology. The AIC value was the minimum for G=3 groups, also pointing out that the three-group model was the best option to describe the data. The evolution and improvement of the AIC values for the different number of groups from G=1 to G=3 are shown in Table 3. Second, if other two-step techniques are used, and in particular a nonhierarchical clustering such as K-means, the number of clusters need to be specified at the beginning. After inspection of the AIC values ($AIC_{k-means}$) when varying the number of groups from 1 through 3, the best solution is also G=3. However, as one may expect, the group membership configuration provided by the EBIVB method outperforms clustering obtained by K-means; thus, it offers a better model for any value of *G*.

In the EBIVB method, the three groups comprise 48.10%, 11.39%, and 40.51% of the sample, respectively. In contrast to these percentages, K-means proportions are approximately 53.16%, 27.85%, and 18.99%, respectively. The results indicate that the proposed algorithm obtained two groups for observations registered prior to 1969 (without following definite temporal classification), whereas the last group comprised observations from 1969 to 2000. Instead, the K-means approach suggested two structural breaks in 1963 and 1985, which split the sample into three groups. Nonetheless, the economic implications of these findings go beyond the scope of this paper.

## 5. Conclusions

Aggregate analysis yields to incorrect results when estimating simultaneous equation models in the presence of considerable heterogeneity in the data. Traditional sequential approaches that estimate separate models for distinct clusters obtained either by a priori assignment or via a clustering method such as K-means may also lead to unsatisfactory outcomes. An alternative two-step strategy for handling heterogeneity in the SEM context was introduced. An incremental variational Bayes clustering algorithm and multigroup simultaneous equation model methodology were combined to study the structural variations of the model. The main advantage of the proposed procedure is that the estimation algorithm does not need any reruns for determining the optimal number of groups and obtaining the clustering of the dataset. The number of groups does not need to be specified a priori, and as a novelty, the groups are formed on the basis of the Gaussian deficiency.

To assess the goodness of fit of this approach, a percentage model was included in the simulation experiments. The study highlighted the good reliability of the EBIVB algorithm in the identification and classification of the different clusters, with performance that was equivalent to a percentage model with *p* lower than 5%. Additionally, the Akaike information criteria of the two-step method reinforced the use of this option over other estimated models. Because of the variety of statistical criteria available for model selection, alternative information heuristics (e.g., CAIC and BIC) need to be examined, and other appealing global measures of fit such as entropy must be explored.

Although the EBIVB algorithm showed good behaviour in the experimental study, its properties could underperform in other situations with ill-conditioned data. For example, estimation problems may arise in the presence of substantially overlapping clusters or large numbers of endogenous variables. The main shortcomings may appear in pattern recognition when finding the prior distribution and mixing coefficients of the Bayesian method. Despite such potential limitations, the suggested procedure is preferable to other traditional approaches for estimating simultaneous equation models when struggling with heterogeneous observations.

## Figures and Tables

**Table 1:** Akaike information criterion (AIC) mean value for different estimated models over s=10 simulation runs and clustering classification error rate committed by the algorithm regarding known group membership model. Note: GM, group membership; PM, percentage model; AGG, aggregate model; CA, proposed clustering algorithm.

Size	GM	PM			AGG	CA	CA Clustering Error
m	p=0%	p=5%	p=10%	p=15%	Aggregate		%
2	76,003.15	76,069.52	76,200.78	76,601.07	82,421.45	75,964.97	1.65
4	144,038.25	148,422.05	150,889.29	152,474.46	153,744.05	144,130.05	0.77
6	238,407.47	244,234.13	247,418.12	248,074.44	249,785.10	238,794.03	0.67
8	332,163.59	338,568.06	343,189.90	344,504.72	349,888.61	333,404.96	0.39

**Table 2:** Evolution of AIC mean value for different estimated models.

Size	Number of Clusters			
m	1	2	3	4
2	83,795.08	78,139.16	76,307.94	75,408.18
4	136,575.21	136,703.96	135,996.17	132,493.36
6	255,276.91	256,088.06	251,367.12	249,979.87
8	348,972.97	351,896.28	341,170.68	331,409.30

**Table 3:** Statistical Criteria for Model Selection.

Number of Clusters	*AIC_k-means_*	*AIC_CA_*
1	2004.415	2004.415
2	1856.893	1855.913
3	1811.696	1638.756

## Data Availability

The data presented in this study are available in Carnero, B. S., Seriñán, P. R., & García, M. M. (2002). El Modelo Klein I y los ciclos económicos. [Klein’s Model I and economic cycles]. Review on Economic Cycles, 4(1).
